# Reliability and Validity Issues Related to Interactive Tailored Patient Assessments: A Case Study

**DOI:** 10.2196/jmir.9.3.e22

**Published:** 2007-08-01

**Authors:** Cornelia M Ruland, Suzanne Bakken, Jo Røislien

**Affiliations:** ^5^Department of BiostatisticsFaculty of MedicineUniversity of OsloOsloNorway; ^4^BiostatisticsRikshospitalet Medical CenterOsloNorway; ^3^Department of Biomedical InformaticsColumbia UniversityNew YorkNYUSA; ^2^Faculty of MedicineUniversity of OsloOsloNorway; ^1^Center for Shared Decision Making and Nursing ResearchRikshospitalet Medical CenterOsloNorway

**Keywords:** Validity, reliability, interactive tailored patient assessments

## Abstract

Recently there has been a proliferation of interactive tailored patient assessment (ITPA) tools. However, evidence of the reliability and validity of these instruments is often missing, which makes their value in research studies questionable. Because several of the common methods to evaluate instrument reliability and validity are not applicable to interactive tailored patient assessments, informatics researchers may benefit from some guidance on which methods of reliability and validity assessment they can appropriately use. This paper describes the main differences between interactive tailored patient assessments and assessment instruments based on psychometric, or classical test, theory; it summarizes the measurement techniques normally used to ascertain the validity and reliability of assessment instruments based on psychometric theory; it discusses which methods are appropriate for interactive tailored patient assessments and which are not; and finally, it illustrates the application of some of the feasible techniques with a case study that describes how the reliability and validity of the tailored symptom assessment instrument called Choice were evaluated.

## Introduction

Recent years have seen a proliferation of interactive health communication tools, together with a growing trend toward empowering patients to take a more active role in their own health care. A prerequisite to effectively helping patients in need of care is to elicit their symptoms and health problems from their perspective. Interactive tailored patient assessments (ITPAs) have become increasingly important as a means of eliciting patients’ illness experiences and tailoring patient care or self-care recommendations to each patient’s individual needs. The ease of deployment of Web-based surveys has made the use of interactive tailored questionnaires more common, and software that allows researchers to rapidly develop custom-tailored questionnaires has started to emerge.

Interactive tailored patient assessments have a number of advantages compared with standardized assessments, in which respondents are required to complete all questions. In interactive tailored patient assessments, the questions can be tailored to each patient individually based on his or her initial responses. Superfluous questions are eliminated, and the questions that remain are more relevant to the patient. For example, the Dialogix system developed at Columbia University implements structured interviews on a series of Web pages. It supports complex branching and conditional tailoring so that questions and summary reports can be tailored to the subject’s responses [1]. The system has been used for surveys on children in the community, the diagnosis of sleep disorder, and depression. Another example is the Composite International Diagnostic Interview (CIDI) used for assessment of mental health disorders, in which positive responses to symptom questions are followed up by other questions, while negative responses often lead to subsequent questions being skipped [2].

One might argue that there do exist traditional assessments that behave somewhat like interactive tailored patient assessments; for example, “if you answered ‘yes’ to question 4, skip questions 5 through 8,” and so on. However, in assessments of this type, *most* questions are answered by all respondents, with additional information gathered for selected subgroups. In interactive tailored patient assessments, however, anything goes; responding, or not, to *any* item can be totally up to the respondent, effectively resulting in each patient completing a “different” assessment. For example, patients can branch into sections that focus on their specific symptoms and problems without being bothered by other questions that are not relevant to them. Because patients complete only a subset of the total number of items available, the response burden is decreased. Consequently, interactive tailored patient assessments allow for an expansion in the breadth and depth of the assessment that helps patients find a closer match between symptom or problem descriptions and their actual illness experiences.

The credibility of interactive tailored patient assessments depends on their ability to adequately capture patients’ experienced symptoms and problems. Validity and reliability are, therefore, crucial issues. Despite an increasing number of studies that use interactive tailored patient assessments as research tools, even in randomized controlled trials, information about reliability and validity is often missing. Consequently, those wishing to implement a specific interactive tailored patient assessment in practice have little assurance about the instrument’s reliability and validity. Also, without such evidence, it is difficult to disseminate study results outside the informatics community and into the clinical literature where a minimum standard for reporting reliability and validity is required for publication. A minimal standard for research instruments should at least include test results of one type of reliability for the group being tested, one type of content validity, and at least one type of criterion-related or construct validity [3].

Psychometric theory offers a number of techniques to examine the reliability and validity of research instruments. However, many of these techniques only apply to instances in which individuals respond to the same set of items, in contrast to interactive tailored patient assessments, in which each informant responds to a different subset of individually selected items. Thus, informatics researchers who are interested in developing an interactive tailored patient assessment are left with the question of which methods they can appropriately use to establish its reliability and validity.

The purpose of this paper is to provide some guidance on evaluating reliability and validity of interactive tailored patient assessments. In it, we (1) describe the main differences between interactive tailored patient assessments and assessment instruments based on classical test theory, using a tailored symptom assessment instrument called Choice as an example,(2) summarize the psychometric techniques normally used to ascertain the validity and reliability of instruments for self-reported assessments, (3) discuss which methods are appropriate for interactive tailored patient assessments and which are not, and finally, (4) illustrate the application of some of the feasible techniques with a case study that describes measurement of the reliability and validity of the Choice instrument. This may serve as a model for other researchers for evaluating reliability and validity of interactive tailored patient assessments.

## Example of an Interactive Tailored Patient Assessment: The Choice Assessment

Choice is the name of a suite of tailored symptom assessment tools designed to help patients report their experienced symptoms and health problems so that their care providers can tailor patient care to each patients’ individual symptoms, problems, and needs. The Choice application used here as an example targets patients with chronic and serious long-term illnesses such as cancer. However, interactive tailored patient assessments are also applicable to other patient populations.

The application is contained and administered via a tablet computer with a touch-sensitive screen or is administered via an Internet application. It supports complex branching, so only relevant questions are asked, and conditional tailoring, so questions are tailored to a subject’s previous responses. For example, in the Choice cancer module, patients first identify among 19 problem categories those that apply to themselves. This triggers a subset of related symptoms from which patients again only select those that apply. For example, if patients initially select the “Problems with eating and drinking” category, they are presented with a more detailed list that helps them specify their eating and drinking problems (eg, taste changes, lack of appetite). The patients then rate the degree of bother and their priorities for care for the selected symptoms. When they are done, the system creates an assessment summary that displays patients’ selected symptoms ranked by their priorities for care. This summary can be used by patients and clinicians for subsequent shared care planning. The Choice instrument has consistently been demonstrated to significantly increase congruence between patients’ reported symptoms and patient care in both rehabilitation and cancer patients [4-6].

## Main Differences Between Interactive Tailored Patient Assessments and Traditional Instruments

### Traditional Instruments

Interactive tailored patient assessments such as the Choice instrument are different in several respects from other standardized measurement approaches that rely on patient self-report. The primary goal of traditional instruments is to support research, that is, to describe, contrast, or compare populations and to arrive at more generalizable conclusions based on specific observations [7]. Instruments can be scales or subscales that are composed of theoretically homogeneous items and that measure an internally consistent construct (eg, depression). Scales meet the criteria of classical test theory, and reliability and validity assessments that are based on measures of internal consistency are appropriate. Another type of instrument is an index, which consists of items that are not necessarily correlated and that together compose the index (eg, a measure of quality of life). Rather than being indicators of the underlying theoretical construct, as in scales, items of an index themselves define the construct. Indexes do not meet assumptions of classical test theory, and internal consistency is, therefore, not a good estimate of reliability or validity [8]. Interactive tailored patient assessments are similar to indexes and, therefore, the same statistical limitations apply.

In the application of either scales or indexes, all respondents complete a given set or subset of items [8,9]. This naturally limits the total number of items that can be, or preferably, should be, contained in an instrument. An indicator of a “good” instrument is parsimony—the instrument’s ability to explain the greatest amount of variance of the concept being measured with the fewest number of items [7]. Given that there is evidence of the reliability and validity of the instrument, higher and lower scores represent higher and lower presence, respectively, of the concept being measured.

### Interactive Tailored Patient Assessments

Interactive tailored patient assessments are primarily designed for clinical application. Thus, the main focus of interest is to elicit characteristics that are unique to a particular person. The purpose is to provide the person with individually tailored care, information, or behavioral change strategies [10]. This is different from the “one size fits all” approach of traditional measurement instruments, in which the focus of interest is the characteristics of populations rather than the individual.

Another difference from traditional assessment instruments is that an interactive tailored patient assessment may be purposely designed to capture each patient’s personal experience. For example, in the Choice instrument, the goal is to help patients find descriptions of their symptoms and health problems that reflect their personal experiences as closely as possible. Thus, patients may choose between relatively similar symptoms that are expressed with synonymous terms, selecting those that they feel are closest to their experience. Such comprehensiveness of symptom descriptions would be difficult in traditional measurement instruments with a parsimonious set of items and would be considered redundant.

There may also be differences in how questions in the instrument are organized and structured. For example, scales combine items into internally consistent scales, or subscales, which tap the same underlying concept. An example is the Center for Epidemiological Studies Depression Scale (CES-D), described later in the case study, which consists of four subscales for which indicators of depression include “problems concentrating” and “sleeping problems” [11]. However, laypersons may not necessarily understand the associations between these two symptoms and depression. To ensure that patients can branch into their symptoms and health problems without difficulty, items in an interactive tailored patient assessment may be grouped according to laypersons’ knowledge structure rather than according to a theoretical concept such as depression. For example, in the Choice instrument, items are organized based on insights gained from systematic investigations of how laypersons organize and label problems and symptoms into meaningful groups [12]. While such a structure supports patient comprehension and recognition, it does not necessarily fit the structure of an internally consistent scale.

Table 1 summarizes differences between traditional measurement instruments and interactive tailored patient assessments.

**Table 1:** Differences Between Traditional Measurement Instruments and Interactive Tailored Patient Assessments

	**Traditional Measurement Instruments**	**ITPA Example: Choice**
**Focus of Interest**	Understanding characteristics of populations; generalizability	Understanding characteristics of individuals
**Primary Purpose**	Research	Clinical practice; to tailor patient care / advice to each individual
**Scale**	1. Each subscale measures one latent concept at a time. Different concepts are contained in internally consistent subscales. 2. Items of an index serve as causal indicators that define the concept being measured.	May capture patients’ symptom and problem experiences on different dimensions Concepts are not necessarily structured into internally consistent subscales, but are organized to fit the patients’ “lay” knowledge structures.
**Set of Questions**	Every respondent completes more or less the same set of questions.	Every respondent completes a different set of questions, based on initial item selection.
**Goal**	Parsimony: to explain the greatest amount of variance in the concept measured with the fewest numbers of items.	Comprehensiveness: to help patients find a close match between the item description and their actual experience.

## Techniques to Measure Reliability and Validity and Their Applicability to Interactive Tailored Patient Assessments

Measurement is the process of linking abstract concepts to empirical indicators. This can happen in two ways. The first is by focusing on the crucial relationship between the observable response and the underlying unobservable theoretical concept. This is the case with concepts such as “intelligence,” which we cannot observe directly, but implications of it, such as peoples’ vocabulary, mathematical ability, and knowledge about the world, stem from this quality. Instruments constructed to capture such concepts have come to be called scales [8,9]. The other possibility is that the unobservable theoretical concept under study is the response to observable explanatory factors. This is the case with, for example, socioeconomic status, which is a function of, say, income and level of education, not the other way around. Instruments constructed to capture such concepts are called indexes, as described earlier [8,9]. The choice of the specific items is much more important in the construction of indexes than of scales.

Reliability and validity are the two basic properties of empirical measurements. Reliability concerns the extent to which an experiment, test, or any measuring procedure yields the same results on repeated trials. Validity is the degree to which an instrument measures what it purports to measure. Reliability is a necessary but not a sufficient condition for validity [13,14]. While reliability and validity are equally important for interactive tailored patient assessments as for other standardized assessments, not all common techniques for measuring reliability and validity are appropriate for interactive tailored patient assessments (Table 2).

**Table 2:** Psychometric concepts, definitions, and methods

**Psychometric Concept**	**Definition**	**Methods**	**Appropriateness for ITPAs**
**Reliability**			
Internal consistency	Average intercorrelation among items	Cronbach alpha, split-half	Inappropriate due to highly variable number of assessment items among respondents
Test-retest	Association between measurements on the same respondents at multiple points in time using the same version of the measurement instrument; coefficient of stability	Correlation between two measurements	Inappropriate if concept being measured changes over time; otherwise appropriate. Even small changes over time might fundamentally change the patient’s response to the interactive tailored patient assessment.
Alternate forms	Association between measurements on the same respondents at multiple points in time using two forms of the “same” measurement instrument; coefficient of equivalence	Correlation between two measurements	Inappropriate if concept being measured changes over time; otherwise appropriate. Due to the nature of the interactive tailored patient assessment, with possibly detailed items, coming up with an alternate form might be difficult.
**Validity**			
Content	Extent to which a specific measure depicts a domain of content	Literature review, expert review	Appropriate
Criterion-related	Extent of correlation between the test and the criterion	Concurrent validity (test and criterion at same point in time); predictive (test and criterion at a future point in time)	Appropriate. Be aware that it might be difficult to find a sensible criterion when many issues are addressed simultaneously, as often is the case.
Construct	Extent to which a particular measure performs in accordance with theoretically derived hypotheses concerning the concepts (or constructs) being measured	Factor analysis, convergent validation, discriminant validation, known group differences, multitrait-multimethod matrix	Factor analysis is often inappropriate due to variable number of assessment items among respondents, or the large sample size that otherwise would be required. Other methods are usually appropriate.

### Measures of Reliability

Common approaches to examine reliability include test-retest, alternate forms, split-half, and tests of internal consistency [13,15].

In the test-retest method, the same test is given to the same people after a period of time [13]. The correlations between the scores in the two administrations of the same test are calculated, and the correlation between two parallel measures equals the reliability coefficient. A prerequisite for test-retest reliability is that the second administration be conducted within a small enough time frame so that the concept being measured (eg, pain) does not change. This is, however, often a problem. Test-retest reliability is appropriate for traditional assessments as well as for interactive tailored patient assessments that measure stable traits, but it is inappropriate for assessments of volatile concepts that change rapidly over time (eg, how bothersome a symptom is).

The alternate form method requires two testing situations with the same people, but an alternate form of the same test is administered [13]. The two forms are intended to measure the same concept. The correlation between the alternative forms provides the estimate of reliability. Similar to the test-retest method, the alternate form of the instrument must be given within a small enough time frame so that the concept being measured has not changed. Under these conditions, the alternate form approach can be appropriate for interactive tailored patient assessments.

In the split-half technique, items of the scale are split in two. To obtain a measure of reliability, the scores of the halves are correlated. This follows the same logic as in the test-retest technique, where the correlation between two parallel measures equals the reliability coefficient. The issue of how to split the items in half, however, is not clear cut.

By far the most popular approach is the internal consistency reliability coefficient Cronbach alpha [16]. Among the reasons for its popularity is the fact that it, like the split-half technique, requires only a single test administration. It does, however, expand on that methodology of the split-half technique, and the calculation of alpha is based on the inter-item correlations among all the items of the scale. The higher the alpha, the higher the reliability [13].

A problem with all the above measures is that they indirectly depend on all respondents completing more or less the same consistent set of items, making the measures difficult to apply to interactive tailored patient assessments. A scale’s reliability is mainly addressed by looking at correlations— mathematical expressions of association. The calculations are done by pairing data and comparing whether variable values behave in a similar manner; if the value of one variable goes up, and the value of another tends to do so as well, the two variables will be more correlated than if this was not so. Problems arise, however, in the presence of missing data (ie, there is no value for a given variable to compare with another). Usually, the issue of missing values in a data set constitutes no major problem when calculating correlations. For example, for 100 patients measured on weight and shoe size, with two persons missing out on the weighting because they were in the gym, this still leaves 98 people for the calculation of the correlation between weight and shoe size for that group of patients. Generally, the amount of missing data in reviewing scales is negligible. There will most likely be some patients that have not answered one item or another, but the amount of pairs left for correlation calculations is rarely affected to such an extent that these calculations suffer severely.

In interactive tailored patient assessments, however, the amount of missing data could be devastatingly high, effectively making well-known techniques useless. Take the Choice instrument. It has a total of 141 symptoms that the patients can choose from. In the testing of the system, the average number of symptoms the patients reported was 10 [17]. That is, for every patient, the average amount of “missing data” after an assessment was more than 90%. Note that these non-answers are actual missing data in the definition of the term: if a patient has not chosen to say something about symptom A, it is not the same as having reported “no bother with symptom A,” which would give a zero value (or similar measure of “nothing”) to use in calculations. But here we do not have any information about how the patient felt about symptom A at the time. Maybe the patient actually had something to say about symptom A but prioritized other items which were more important or simply forgot to respond to that item.

This lack of a fixed system of items to perform calculations on in order to verify the reliability of an interactive tailored patient assessment constitutes a major statistical challenge. All correlation calculations are deemed to be suffering from this fact, and all correlations will be calculated less precisely since the unanswered questions will contribute a “missing,” erasing that piece of information totally, rather than a zero or similar value, as in more traditional assessments. For example, a patient answering items 1 through 5 in one administration of an interactive tailored patient assessment and items 2 through 10 in another administration of the same interactive tailored patient assessment, would, in a test-retest, only have four items in common for the two administrations, even though five items were answered the first time and nine the second time, for a total of 10 different items.

The calculation of Cronbach alpha [16] depends on the number of items and the mean inter-item correlation. For interactive tailored patient assessments, however, one needs an adjustment for the fact that each patient only responds to a small subdomain of *N*, which will differ from patient to patient. Further, the inter-item correlation is then based on an extremely sparsely filled scale. Finally, the shared size of the interactive tailored patient assessment instrument is a possible problem in itself; with 100 items, an average inter-item correlation of only 0.04 is enough to ensure an alpha of .80.

Factor analysis is closely linked to reliability measures, but makes less stringent assumptions than alpha-type methods. Such methods are, however, also deemed to be unreliable in the setting described above. Factor analysis does nothing more than redefine and simplify the correlation matrix, a matrix that may be calculated on the basis of a huge amount of missing data and very sparse real information. The number of assessments needed in order to have a trustworthy correlation matrix would then have to be extremely high. There are several guidelines for sample size. Among others, Tinsley and Tinsley [18] suggest a ratio of 5-10 subjects per item, up to about 300 subjects. Thereafter, the ratio can be somewhat relaxed. Comrey [19], on the other hand, stated that a sample size of 200 is adequate in most cases of ordinary factor analysis that involve no more than 40 items. However, this calculation breaks down for a 141-item assessment in which each individual selects approximately 10 items; the exact sample size needed in these instances thus becomes very difficult to calculate. Cronbach alpha and other similar measures, as well as factor analysis methodologies, are indirectly based on the fact that all patients fill out the same fixed set of items or close thereto. To our knowledge, nobody has refined these statistical measures to cope with the problems described above. Validating interactive tailored patient assessments thus relies on carefully reviewing the options at hand to see whether they will be applicable for a given instrument. For the Choice instrument, a hybrid of test-retest and alternate forms was used for reliability assessment. It is described in more detail below in the case study.

### Measures of Validity

The main methods to assess the validity of a test for a group of people under certain circumstances are content validity, criterion-related validity, and construct validity. Fundamentally, content validity depends on the extent to which an empirical measurement reflects a specific domain of content and whether the items reflect the meaning associated with each dimension or subdimension [13] of that measure. Content validity is crucial for all measurements, including interactive tailored patient assessments, but unfortunately there is no rigorous way to assess it [13].

Criterion-related validity refers to the correlation of a measure with a criterion variable that is external to the measuring instrument itself [15]. The higher the correlation, the more valid is the measure for the particular criterion. The measurements may be collected at the same point in time (concurrent validity), or the measurement under study may be used to predict a future measurement (predictive validity). For example, the degree to which a test for college admission can predict later academic achievement reflects criterion-related validity of the test. The availability of a criterion measurement (ie, a gold standard) is a prerequisite to examining criterion-related validity of any assessment, tailored or untailored. Because such a gold standard is often missing, measuring criterion-related validity is difficult.

In contrast to content validity and criterion-related validity, construct validity has a more generalized applicability and lends itself easier to empirical investigation. Constructs concern domains of variables [15]. Construct validity is concerned with the extent to which a particular measure relates to other measurements consistent with theoretically derived hypotheses concerning the construct being measured [13]. There are three major aspects of construct validation: (1) specifying the domain of observables related to the construct, (2) determining the extent to which observables measure the same thing, and (3) performing subsequent experiments to determine the extent to which supposed measures of the construct are consistent with “best guesses” about the construct [7,15].

A number of techniques for examining construct validity are applicable to interactive tailored patient assessments. For example, convergent and discriminant approaches, including known group differences, are based on hypothesized relationships between the measurement of concern and another variable. Convergent validity is demonstrated when two independent methods that measure the same variable or attribute are highly correlated. Divergent validity is demonstrated when measures of different attributes do not highly correlate.

In their seminal paper on construct validation, Campbell and Fiske [20] proposed the multitrait-multimethod matrix as an approach to examining convergent and discriminant validity. The multitrait-multimethod matrix includes two traits (one of primary interest) and two methods that are applied to both traits. The basic premise is that the measurements of a trait will converge across methods and diverge between traits. For example, measurements related to dyspnea severity should converge across paper-based and computer-based assessment methods, but the measurement of dyspnea severity should be less highly correlated with the measurement of nausea severity using the same method.

Other techniques to establish construct validity that examine the internal structure of a measurement instrument, such as factor analysis, are, however, often inappropriate for interactive tailored patient assessments because of their dependence on a reliable correlation matrix. The share size of the population needed to verify the instrument, coping with both the possible three-digit number of items and the possible close-to-100% missing data, could approach numbers way out of practical reach. Table 2 summarizes psychometric concepts, measurement methods, and their appropriateness for interactive tailored patient assessments.

## A Case Study: Examining Reliability and Validity of the Choice Instrument

### Reliability Assessment

When testing the reliability of Choice, it was evident that we needed a way of being able to pair observations on the different items without encountering an overwhelming amount of missing data. Because questions in the Choice instrument are tailored to each respondent based on initial response, reliability measures that are built on internal consistency could not be appropriately used for the evaluation of reliability.

A first thought was to perform a test-retest, as it would be natural to assume that an individual would correlate higher with himself or herself (ie, having the same bothersome symptoms and same priorities for treatment if the time frame between the tests was sufficiently short), reducing the amount of missing data in the correlation pairing. A complete test-retest using the Choice instrument felt inappropriate, however, because of the risk that patients’ symptom reports could change to such an extent that the discrepancy between items chosen in the test and the retest would make the correlation calculations unreliable. This concern was strengthened by the fact that several of the items address issues that change fairly quickly with time.

The alternate form approach seemed a logical second option, but as the Choice instrument contains 141 symptoms with several nuances in the wording to capture the specific disease pattern of the particular patient, as described earlier, an alternate form could run the risk of being different in such a way that patients would choose other symptoms merely due to the wording of the items. It seemed difficult to come up with an acceptable, completely alternative form of the instrument. There did, however, exist a somewhat alternative format of the Choice instrument that would at the same time minimize the amount of missing data: the full list of the 141 symptoms. We used this to assess the reliability of the Choice instrument.

To collect the reliability data, we conducted a separate study independent from our clinical trial. Because reliability is sample-specific, patients in this new study were recruited from the same population and setting and had to meet the same inclusion criteria as patients in the clinical trial. After Institutional Review Board approval was obtained, 100 patients undergoing cancer treatment were recruited. First, patients were asked to complete the tailored Choice assessment similar to patients in the clinical trial. Immediately after and in the same data collection session, they were asked to complete a questionnaire, the alternate form that included the full set of 141 symptom descriptions contained in Choice. The correlation between Choice and questionnaire data was 0.74 for all symptoms, and 0.85 for moderately or very bothersome symptoms [17]. According to Nunnally and Bernstein, correlations greater than 0.70 provide evidence of the satisfactory reliability of a measurement instrument [15].

It may at first be surprising that the correlation coefficients between the two formats were not higher. The main reason was that in the Choice instrument it is possible to choose different terms to express almost the same symptom. For example, a patient who chose “lack of energy” in the interactive tailored patient assessment version, chose instead “fatigued” in the paper-based form. While the patient may not have been aware of this distinction, this weakened the correlations between the two forms, making them somewhat lower than one might expect.

### Validity Assessment

#### Content Validity

As above mentioned, content validity depends greatly on the adequacy with which a specific domain of content is sampled [15]. While this is difficult to measure directly, thorough and appropriate procedures used during the development of a new instrument are a prerequisite of content validity. It is impossible to specify exactly how many items need to be developed for a particular domain of content. However, it is always preferable to initially create too many items rather than too few as inadequate items can always be eliminated [13]. This is particularly true for interactive tailored patient assessments, in which patients complete only those subsets of items relevant to them, and the total number of items thus matters less. Here we describe the process for developing and ensuring content validity of the Choice module for cancer patients.

The goal when constructing the tailored Choice instrument was to assist patients in communicating their illness experience along physical, psychosocial, and functional dimensions as close as possible to their actual experiences. It was, therefore, important to include a comprehensive set of items that reflected all dimensions of patients’ illness experiences in sufficient level of detail and that were expressed in lay language to support patient recognition and communication.

To identify items to be included, we conducted a thorough review of the scientific literature to identify problems, specific symptoms, and functional limitations encountered by cancer patients. This search and review included the health care bibliographic databases as well as the World Wide Web and resulted in a preliminary list of symptoms and functional problems for potential inclusion. Expert groups of specialists in cancer care (physicians, nurses, social workers) then critically reviewed this list for relevance, comprehensibility, completeness, and level of detail and supplemented it with expert opinion [6]. Particular attention was paid to expressing symptoms and problems in simple, understandable, nonmedical lay language. Next, the revised symptom list was presented to 15 cancer inpatients and outpatients (9 women, 6 men; age 40-74 years) who were asked to complete and evaluate a paper-based version of the symptom assessment for clarity of meaning, appropriateness, wording, completeness, redundancy, and format, and to add comments. This resulted in further suggestions for revisions, which were discussed in the cancer expert groups. The subsequently refined symptom list was then implemented in the tailored computer application and pilot tested with 56 outpatients with varying cancer diagnoses [6]. Based on this pilot study, a few item descriptions were revised to better describe symptoms from the perspective of the patients. The final version was used for the reliability testing described above and in the clinical trial that provided data for the validity testing described below.

#### Construct Validity

To evaluate construct validity of the Choice instrument, we used known group differences techniques as well as assessments of convergent and discriminant validity. We performed three evaluations of known group differences based on data collected in a clinical trial of 148 patients who received active cancer treatment for leukemia and lymphoma.

The first test was based on the hypothesis that patients undergoing a stem cell transplant would report more symptoms with the Choice instrument than patients treated with chemotherapy only. This hypothesis is consistent with empirical evidence on treatment side effects and was supported by the data. Patients undergoing a stem cell transplant reported significantly more symptoms than patients in the chemotherapy group (14.6 vs 9.2, *P* < .001).

In the second test, we examined gender differences in self-reported symptoms. Because the literature has provided some evidence that women report more symptoms than men [21], we expected that this difference would also be found with the Choice instrument. This was again supported. In our clinical trial, women reported significantly more symptoms than men (13.7 vs 10.0, *P* < .001).

Finally, we examined whether the most reported symptoms during patients’ illness trajectories were consistent with expected symptom patterns during different phases of treatment and rehabilitation. This was again supported. The most frequently selected symptoms 1 to 2 months into treatment were side effects related to chemotherapy and stem cell transplant, including nausea, vomiting, and mouth sores. During the third and fourth months of treatment, long-term side effects such as neurological problems, memory problems, and weight loss started to occur more frequently. During rehabilitation, the number of physical symptoms decreased and the focus of self-reported symptoms shifted to issues regarding resuming a normal life and worries about the future. Thus, all three known group difference tests performed as expected and provided support for the validity of the Choice instrument.

To measure convergent and discriminant validity, we compared the performance of the Choice instrument in our clinical trial data set with two other measures taken at the same time point: the CES-D [11] and the SF-36, a multidimensional measure of health-related quality of life [22]. Ideally, measures of a similar trait should correlate higher with each other than they do with measures of different traits. To estimate convergent validity, we computed the correlations between the psychosocial subscales of the Choice instrument and both the CES-D depression subscale and the SF-36 mental health index subscale. A correlation of 0.57 was found with the CES-D depression subscale and −0.64 with the SF-36 mental health index. Similar evidence of convergent validity was found for physical symptoms. The physical symptom subscales of the Choice instrument strongly correlated with the SF-36 bodily pain scale (*r* = −0.61), the SF-36 physical health component subscale (*r* = −0.54), and the SF-36 physical functioning subscale (*r* = −0.44).

To assess discriminant validity, we performed correlations between Choice subscales and CES-D and SF-36 subscales that measured different attributes, hypothesizing that they would not correlate to a very high degree. This was supported by our data. The physical symptom subscales of the Choice instrument correlated only weakly with the CES-D depression subscale (*r* = 0.25) and the SF-36 mental health index (*r* = −0.28). Similarly, psychosocial symptoms in the Choice instrument correlated weakly with the SF-36 physical functioning subscale (*r* = −0.18) and the physical health component subscale (*r* = −0.13).

## Conclusion

In this paper, we strongly advocate evaluating and reporting reliability and validity of interactive tailored patient assessments, which is crucial for the credibility of interactive tailored patient assessments as research instruments. However, several of the common measurement techniques available to assess these psychometric properties are not applicable to interactive tailored patient assessments. The advantage of computerized tailored assessments is that patients can skip unimportant items and hone in on problems that matter to them and that reflect their actual experience. However, this advantage makes reliability and validity assessments of interactive tailored patient assessments a challenge for informatics researchers. To assist in this task, we have discussed which techniques might be feasible for establishing reliability and validity of interactive tailored patient assessments and demonstrated their application in a case study of the Choice instrument.

Although assessment of reliability of an interactive tailored patient assessment may require collection of a separate data set in addition to the clinical trial data, this is well worth the effort. A basic core of evidence of reliability and validity is needed for any instrument. Reliability is a prerequisite for validity, and an unreliable instrument cannot be valid. Unreliable and invalid instruments are not worth further investigation [3]. Reporting of interactive tailored patient assessment reliability and validity should become a requirement for publishable informatics research, so researchers can trust the data. Evidence of reliability and validity has long been a requirement for publication of research instruments in the clinical literature, and is, therefore, a prerequisite for the dissemination of informatics tools outside the informatics community. The adoption of a similar requirement in scientific informatics journals would greatly enhance the state of science in the field of tailored assessments and health interventions.

## Abbreviations

CES-D: Center for Epidemiological Studies Depression Scale
ITPA: interactive tailored patient assessment
SF-36: Medical Outcomes Study 36-Item Short Form Health Survey
